# Supplementary material for: A Likelihood Approach for Real-Time Calibration of Stochastic Compartmental Epidemic Models
Source: PLoS Comput Biol. 2017 Jan 17;13(1):e1005257. doi: 10.1371/journal.pcbi.1005257 (PMC5240920; doi:10.1371/journal.pcbi.1005257)
Supplement: S1 File — (TAR.GZ) [file pcbi.1005257.s014.tar.gz › HSPH_Online-SI-Revision/IPoi09/n1-extreme/n1-extreme_BENCH2_table0.pdf]

1	newly 3 weeks spec	79.	80.1094	{46., 121.}	29.2919	0.130067	newly 3 weeks cumul	342.	287.625	{207., 362.}	18.7226	0.0969645	newly 5 weeks spec	40.	46.6406	{22., 79.}	43.1641	0.16277	newly 5 weeks cumul	434.	396.641	{264., 534.}	20.4025	0.0991351	newly 60 weeks cumul	525.	509.641	{332., 739.}	25.4851	0.11569
2	newly 3 weeks spec	61.	49.2083	{30., 71.}	27.6184	0.151141	newly 3 weeks cumul	211.	185.5	{122., 257.}	22.1301	0.112023	newly 5 weeks spec	24.	29.25	{16., 45.}	44.213	0.163483	newly 5 weeks cumul	275.	252.125	{167., 354.}	23.8838	0.116079	newly 56 weeks cumul	368.	340.986	{211., 509.}	27.2532	0.130801
3	newly 3 weeks spec	93.	77.0417	{49., 100.}	20.5346	0.109047	newly 3 weeks cumul	270.	256.764	{202., 307.}	13.2253	0.0642838	newly 5 weeks spec	51.	52.5972	{31., 78.}	29.2211	0.128303	newly 5 weeks cumul	381.	372.083	{286., 455.}	15.3106	0.0704921	newly 58 weeks cumul	577.	552.319	{394., 735.}	20.299	0.0942412
4	newly 3 weeks spec	61.	72.7403	{49., 99.}	28.3159	0.10873	newly 3 weeks cumul	180.	237.468	{178., 283.}	34.0043	0.124407	newly 5 weeks spec	40.	51.5455	{33., 71.}	38.0195	0.136636	newly 5 weeks cumul	278.	350.195	{256., 420.}	29.8281	0.111288	newly 57 weeks cumul	490.	536.182	{362., 706.}	22.9049	0.0942151
5	newly 3 weeks spec	56.	0.	{0., 0.}	100.	2.74819	newly 3 weeks cumul	216.	0.	{0., 0.}	100.	3.33445	newly 5 weeks spec	40.	0.	{0., 0.}	100.	2.60206	newly 5 weeks cumul	294.	0.	{0., 0.}	100.	3.46835	newly 50 weeks cumul	415.	0.	{0., 0.}	100.	3.61805
6	newly 3 weeks spec	54.	52.6567	{32., 79.}	24.9862	0.116333	newly 3 weeks cumul	153.	184.94	{128., 240.}	28.6021	0.107851	newly 5 weeks spec	31.	36.0746	{20., 61.}	39.8652	0.1546	newly 5 weeks cumul	228.	266.463	{182., 369.}	28.1422	0.10775	newly 55 weeks cumul	355.	406.597	{246., 613.}	32.5205	0.123362
7	newly 3 weeks spec	68.	44.5972	{20., 78.}	43.6479	0.27539	newly 3 weeks cumul	227.	184.042	{107., 285.}	31.0022	0.165851	newly 5 weeks spec	52.	23.0694	{9., 41.}	57.3451	0.422062	newly 5 weeks cumul	341.	239.472	{134., 369.}	37.0723	0.215531	newly 59 weeks cumul	479.	292.389	{153., 445.}	43.679	0.272118
8	newly 3 weeks spec	44.	29.9077	{14., 47.}	42.7972	0.255532	newly 3 weeks cumul	184.	112.508	{62., 178.}	41.2793	0.259917	newly 5 weeks spec	25.	19.2615	{7., 34.}	50.0308	0.29645	newly 5 weeks cumul	249.	156.015	{82., 248.}	42.0884	0.264335	newly 53 weeks cumul	332.	227.846	{111., 366.}	42.8916	0.255757
9	newly 3 weeks spec	65.	62.0556	{36., 85.}	29.0598	0.133385	newly 3 weeks cumul	257.	238.431	{153., 349.}	23.3949	0.111517	newly 5 weeks spec	29.	30.8333	{17., 48.}	40.5172	0.166892	newly 5 weeks cumul	344.	314.597	{188., 463.}	26.3445	0.12589	newly 60 weeks cumul	427.	390.028	{228., 559.}	28.9617	0.137761
10	newly 3 weeks spec	63.	41.9333	{17., 66.}	41.2698	0.259053	newly 3 weeks cumul	193.	144.233	{74., 227.}	32.1071	0.187035	newly 5 weeks spec	45.	31.7167	{11., 58.}	43.5185	0.273006	newly 5 weeks cumul	286.	211.65	{101., 339.}	34.6795	0.203092	newly 53 weeks cumul	451.	350.383	{150., 589.}	41.4375	0.236117
11	newly 3 weeks spec	56.	43.7237	{22., 74.}	38.2284	0.208155	newly 3 weeks cumul	197.	176.868	{102., 261.}	28.1726	0.137807	newly 5 weeks spec	31.	22.5263	{9., 40.}	44.3124	0.261668	newly 5 weeks cumul	268.	229.934	{132., 342.}	30.9554	0.156023	newly 59 weeks cumul	345.	280.566	{155., 418.}	34.5118	0.179983
12	newly 3 weeks spec	60.	90.6563	{59., 121.}	54.1667	0.177403	newly 3 weeks cumul	251.	299.297	{243., 351.}	21.9186	0.0840416	newly 5 weeks spec	36.	59.3281	{31., 97.}	70.7031	0.208849	newly 5 weeks cumul	329.	433.734	{324., 534.}	33.8288	0.121542	newly 60 weeks cumul	420.	597.453	{409., 795.}	44.7284	0.150586
13	newly 3 weeks spec	43.	45.0938	{22., 64.}	30.3052	0.136462	newly 3 weeks cumul	128.	151.828	{91., 202.}	32.312	0.128936	newly 5 weeks spec	32.	34.125	{15., 57.}	40.7227	0.180288	newly 5 weeks cumul	184.	227.109	{128., 323.}	38.018	0.146264	newly 53 weeks cumul	478.	371.828	{191., 569.}	31.6586	0.181212
14	newly 3 weeks spec	35.	50.0667	{26., 90.}	54.3238	0.169631	newly 3 weeks cumul	175.	185.213	{106., 299.}	31.2229	0.13154	newly 5 weeks spec	20.	29.84	{11., 61.}	67.2	0.203053	newly 5 weeks cumul	217.	254.253	{137., 441.}	38.3287	0.14531	newly 57 weeks cumul	250.	348.053	{181., 656.}	53.2907	0.172458
15	newly 3 weeks spec	59.	41.6765	{24., 64.}	32.5025	0.224529	newly 3 weeks cumul	186.	142.544	{79., 195.}	25.5614	0.167358	newly 5 weeks spec	32.	30.9706	{14., 53.}	36.3971	0.204086	newly 5 weeks cumul	268.	210.441	{112., 298.}	26.1962	0.173041	newly 52 weeks cumul	342.	348.706	{163., 584.}	36.0423	0.188749
16	newly 3 weeks spec	56.	44.6944	{21., 70.}	36.2599	0.202039	newly 3 weeks cumul	175.	175.597	{108., 261.}	30.2778	0.133828	newly 5 weeks spec	29.	23.75	{9., 42.}	43.0077	0.237844	newly 5 weeks cumul	242.	232.028	{132., 340.}	32.0707	0.148125	newly 59 weeks cumul	393.	293.972	{154., 439.}	35.8072	0.202262
17	newly 3 weeks spec	48.	66.0417	{40., 95.}	46.6146	0.160297	newly 3 weeks cumul	177.	207.819	{152., 270.}	26.9852	0.110144	newly 5 weeks spec	42.	51.0556	{27., 80.}	43.1217	0.161633	newly 5 weeks cumul	264.	315.611	{218., 411.}	29.1877	0.113428	newly 56 weeks cumul	488.	536.931	{318., 779.}	31.2813	0.127393
18	newly 3 weeks spec	77.	46.8615	{27., 69.}	42.3377	0.261901	newly 3 weeks cumul	277.	182.877	{117., 257.}	35.6568	0.210545	newly 5 weeks spec	51.	26.3692	{13., 42.}	54.3288	0.363044	newly 5 weeks cumul	383.	245.538	{151., 357.}	38.3892	0.230305	newly 59 weeks cumul	532.	313.938	{191., 476.}	44.2337	0.274902
19	newly 3 weeks spec	85.	48.1746	{22., 89.}	47.7311	0.314884	newly 3 weeks cumul	252.	190.444	{115., 313.}	34.3537	0.190708	newly 5 weeks spec	73.	24.4762	{11., 48.}	67.2538	0.556054	newly 5 weeks cumul	405.	251.159	{140., 449.}	42.3986	0.262674	newly 60 weeks cumul	563.	310.143	{169., 543.}	48.3239	0.318312
20	newly 3 weeks spec	46.	43.7917	{20., 75.}	41.6365	0.206116	newly 3 weeks cumul	195.	152.111	{81., 244.}	33.2764	0.188332	newly 5 weeks spec	24.	29.8611	{13., 58.}	59.9537	0.223787	newly 5 weeks cumul	257.	219.347	{113., 368.}	35.3275	0.187083	newly 53 weeks cumul	327.	336.069	{161., 617.}	45.4766	0.201238
21	newly 3 weeks spec	59.	64.3125	{46., 81.}	19.5975	0.0808491	newly 3 weeks cumul	155.	205.594	{162., 247.}	33.4879	0.120735	newly 5 weeks spec	46.	47.8906	{27., 71.}	29.5177	0.128901	newly 5 weeks cumul	257.	309.234	{237., 369.}	24.775	0.0941215	newly 56 weeks cumul	478.	508.359	{337., 714.}	23.8657	0.0997825
22	newly 3 weeks spec	49.	33.6216	{18., 53.}	39.1616	0.231763	newly 3 weeks cumul	172.	136.811	{84., 203.}	31.7096	0.167497	newly 5 weeks spec	32.	18.9595	{9., 31.}	47.2551	0.303343	newly 5 weeks cumul	242.	181.703	{112., 275.}	34.5544	0.187874	newly 57 weeks cumul	358.	236.959	{140., 387.}	41.1558	0.240459
23	newly 3 weeks spec	51.	63.	{38., 85.}	34.2447	0.125348	newly 3 weeks cumul	194.	230.944	{161., 295.}	26.4048	0.100539	newly 5 weeks spec	37.	36.2113	{21., 55.}	31.8234	0.144566	newly 5 weeks cumul	294.	315.535	{216., 412.}	21.8885	0.090739	newly 59 weeks cumul	412.	413.592	{274., 549.}	24.2753	0.106552
24	newly 3 weeks spec	65.	80.8689	{48., 121.}	37.9823	0.1388	newly 3 weeks cumul	306.	289.934	{207., 363.}	17.7328	0.0834644	newly 5 weeks spec	35.	46.9344	{27., 71.}	50.1171	0.164898	newly 5 weeks cumul	386.	399.869	{286., 527.}	22.1099	0.0950772	newly 60 weeks cumul	448.	513.82	{345., 695.}	29.6948	0.1143
25	newly 3 weeks spec	55.	58.662	{38., 75.}	23.201	0.0981123	newly 3 weeks cumul	189.	202.62	{151., 255.}	18.6676	0.0797908	newly 5 weeks spec	45.	43.2958	{23., 69.}	31.831	0.14672	newly 5 weeks cumul	280.	296.169	{212., 384.}	19.5272	0.0826899	newly 57 weeks cumul	488.	468.141	{305., 691.}	25.5715	0.116181
26	newly 3 weeks spec	68.	75.0476	{48., 108.}	28.5247	0.117274	newly 3 weeks cumul	336.	262.238	{186., 337.}	22.6238	0.124471	newly 5 weeks spec	22.	48.8413	{21., 74.}	125.325	0.311138	newly 5 weeks cumul	403.	371.857	{255., 476.}	19.1658	0.0939407	newly 59 weeks cumul	451.	507.365	{323., 710.}	29.3492	0.117501
27	newly 3 weeks spec	39.	42.9437	{21., 71.}	39.0755	0.163069	newly 3 weeks cumul	136.	151.437	{85., 232.}	32.7672	0.133676	newly 5 weeks spec	42.	29.8169	{15., 56.}	43.4272	0.256251	newly 5 weeks cumul	218.	218.141	{117., 351.}	30.8567	0.139198	newly 54 weeks cumul	436.	331.437	{171., 558.}	38.9973	0.218155
28	newly 3 weeks spec	51.	53.913	{29., 94.}	33.9585	0.14109	newly 3 weeks cumul	228.	205.957	{131., 296.}	24.0211	0.116507	newly 5 weeks spec	34.	31.6812	{15., 54.}	42.37	0.195863	newly 5 weeks cumul	314.	279.768	{167., 409.}	27.527	0.134168	newly 58 weeks cumul	442.	359.609	{202., 562.}	33.4219	0.173646
29	newly 3 weeks spec	80.	56.5574	{36., 77.}	32.623	0.183607	newly 3 weeks cumul	289.	192.59	{139., 246.}	33.6434	0.189139	newly 5 weeks spec	36.	40.7541	{22., 72.}	38.2514	0.152533	newly 5 weeks cumul	356.	284.082	{195., 373.}	24.3185	0.128084	newly 55 weeks cumul	420.	457.082	{264., 697.}	29.7502	0.120604
30	newly 3 weeks spec	25.	26.6102	{9., 53.}	49.2881	0.218258	newly 3 weeks cumul	94.	93.322	{33., 166.}	45.1136	0.212725	newly 5 weeks spec	18.	16.9492	{5., 33.}	59.887	0.302176	newly 5 weeks cumul	134.	131.915	{48., 247.}	48.3557	0.227868	newly 50 weeks cumul	194.	205.	{62., 381.}	57.4436	0.249218
31	newly 3 weeks spec	69.	37.1429	{15., 77.}	55.4451	0.37863	newly 3 weeks cumul	249.	152.7	{72., 314.}	46.4314	0.290464	newly 5 weeks spec	42.	19.8143	{7., 37.}	60.3061	0.45028	newly 5 weeks cumul	339.	201.186	{93., 404.}	49.1066	0.312779	newly 59 weeks cumul	419.	248.086	{111., 494.}	51.2513	0.328505
32	newly 3 weeks spec	64.	70.3284	{42., 110.}	34.2351	0.138996	newly 3 weeks cumul	288.	257.746	{174., 340.}	19.8642	0.0986169	newly 5 weeks spec	34.	42.4776	{17., 74.}	53.468	0.195811	newly 5 weeks cumul	366.	356.015	{219., 494.}	23.8847	0.110128	newly 59 weeks cumul	414.	464.791	{269., 734.}	32.5222	0.129323
33	newly 3 weeks spec	77.	53.3088	{31., 78.}	32.9832	0.193244	newly 3 weeks cumul	224.	175.662	{124., 226.}	22.5381	0.122439	newly 5 weeks spec	47.	41.4412	{22., 73.}	34.9812	0.179054	newly 5 weeks cumul	333.	264.265	{171., 350.}	22.7345	0.124885	newly 55 weeks cumul	564.	450.735	{254., 727.}	31.2943	0.171174
34	newly 3 weeks spec	60.	68.96	{42., 93.}	28.4444	0.111777	newly 3 weeks cumul	219.	245.933	{180., 304.}	20.2253	0.0806422	newly 5 weeks spec	38.	46.1467	{25., 75.}	41.8596	0.151531	newly 5 weeks cumul	306.	348.88	{251., 475.}	23.695	0.0928264	newly 58 weeks cumul	433.	482.293	{309., 691.}	27.0208	0.106862
35	newly 3 weeks spec	68.	54.1429	{30., 83.}	32.3529	0.176762	newly 3 weeks cumul	222.	213.057	{138., 290.}	22.4582	0.104767	newly 5 weeks spec	42.	31.1286	{17., 52.}	39.4898	0.218824	newly 5 weeks cumul	317.	285.429	{178., 394.}	24.9301	0.122596	newly 59 weeks cumul	467.	364.429	{212., 533.}	30.5231	0.166091
36	newly 3 weeks spec	69.	60.3088	{37., 91.}	24.8721	0.128437	newly 3 weeks cumul	224.	208.221	{131., 265.}	17.9819	0.0911228	newly 5 weeks spec	46.	43.2353	{20., 73.}	33.8235	0.16502	newly 5 weeks cumul	333.	301.118	{189., 408.}	20.3763	0.101837	newly 56 weeks cumul	462.	463.456	{265., 669.}	27.3141	0.121782
37	newly 3 weeks spec	58.	40.5536	{23., 59.}	32.0505	0.191288	newly 3 weeks cumul	192.	134.286	{82., 194.}	30.9524	0.178676	newly 5 weeks spec	31.	31.3214	{11., 51.}	39.0553	0.183922	newly 5 weeks cumul	273.	201.411	{111., 293.}	29.3498	0.167809	newly 51 weeks cumul	413.	348.714	{176., 616.}	36.5444	0.189064
38	newly 3 weeks spec	60.	47.3279	{34., 70.}	29.3169	0.156148	newly 3 weeks cumul	244.	172.738	{125., 236.}	29.918	0.166911	newly 5 weeks spec	40.	33.5902	{18., 51.}	38.4016	0.19359	newly 5 weeks cumul	319.	247.41	{165., 339.}	27.2419	0.146353	newly 56 weeks cumul	406.	373.902	{230., 524.}	31.0829	0.144679
39	newly 3 weeks spec	93.	60.1806	{37., 86.}	36.1858	0.21272	newly 3 weeks cumul	314.	216.208	{156., 278.}	31.1438	0.174214	newly 5 weeks spec	46.	36.2778	{21., 52.}	33.5749	0.181031	newly 5 weeks cumul	440.	301.181	{212., 409.}	32.3138	0.183073	newly 59 weeks cumul	558.	410.833	{270., 556.}	31.3869	0.173304
40	newly 3 weeks spec	44.	33.0282	{14., 63.}	44.7183	0.257826	newly 3 weeks cumul	165.	131.831	{76., 235.}	37.277	0.199415	newly 5 weeks spec	31.	17.5352	{7., 36.}	56.2472	0.397144	newly 5 weeks cumul	230.	172.817	{91., 315.}	40.6001	0.226785	newly 57 weeks cumul	342.	216.62	{108., 383.}	45.647	0.28379
41	newly 3 weeks spec	54.	41.9643	{24., 61.}	27.7116	0.156791	newly 3 weeks cumul	164.	146.768	{83., 203.}	22.7243	0.121183	newly 5 weeks spec	33.	33.5179	{16., 54.}	33.2792	0.154977	newly 5 weeks cumul	248.	218.429	{124., 298.}	23.3583	0.123854	newly 52 weeks cumul	369.	376.661	{188., 592.}	33.2511	0.15219
42	newly 3 weeks spec	75.	56.3968	{29., 83.}	36.1905	0.201536	newly 3 weeks cumul	284.	220.111	{137., 304.}	27.6492	0.152701	newly 5 weeks spec	55.	29.4127	{13., 45.}	56.6234	0.378799	newly 5 weeks cumul	401.	290.778	{169., 422.}	34.2398	0.193764	newly 60 weeks cumul	505.	360.683	{195., 545.}	37.8626	0.215762
43	newly 3 weeks spec	82.	80.1757	{51., 111.}	24.6374	0.114656	newly 3 weeks cumul	252.	273.811	{209., 341.}	18.7151	0.0770577	newly 5 weeks spec	62.	50.5676	{28., 85.}	34.0889	0.18348	newly 5 weeks cumul	378.	389.689	{271., 504.}	20.2738	0.0882588	newly 59 weeks cumul	545.	535.446	{349., 744.}	24.1185	0.109824
44	newly 3 weeks spec	70.	61.8784	{37., 93.}	27.6255	0.138455	newly 3 weeks cumul	222.	219.027	{147., 297.}	19.6981	0.0892542	newly 5 weeks spec	43.	38.2838	{20., 64.}	33.0924	0.164775	newly 5 weeks cumul	326.	307.892	{202., 427.}	21.7294	0.102406	newly 57 weeks cumul	430.	434.905	{267., 626.}	27.6964	0.121441
45	newly 3 weeks spec	80.	73.4706	{43., 112.}	28.5662	0.137193	newly 3 weeks cumul	274.	274.544	{201., 350.}	18.1462	0.0807054	newly 5 weeks spec	45.	42.5	{20., 72.}	37.5817	0.176968	newly 5 weeks cumul	386.	373.838	{247., 506.}	21.7121	0.0992452	newly 60 weeks cumul	569.	471.529	{290., 674.}	26.7342	0.138794
46	newly 3 weeks spec	54.	50.1842	{27., 82.}	33.5283	0.161915	newly 3 weeks cumul	225.	177.329	{103., 257.}	27.9942	0.152896	newly 5 weeks spec	46.	33.8816	{16., 63.}	42.5915	0.242144	newly 5 weeks cumul	335.	251.816	{144., 404.}	31.5947	0.177902	newly 57 weeks cumul	485.	366.434	{194., 621.}	37.344	0.207091
47	newly 3 weeks spec	69.	76.5352	{53., 95.}	22.5964	0.0909215	newly 3 weeks cumul	202.	255.169	{211., 304.}	27.7158	0.102938	newly 5 weeks spec	59.	52.9718	{35., 72.}	24.8747	0.120918	newly 5 weeks cumul	320.	373.803	{297., 451.}	20.9067	0.081082	newly 58 weeks cumul	544.	555.465	{399., 737.}	19.1332	0.0823628
48	newly 3 weeks spec	53.	54.3889	{33., 81.}	29.4025	0.128441	newly 3 weeks cumul	180.	197.639	{129., 264.}	24.9537	0.102173	newly 5 weeks spec	40.	34.3889	{15., 55.}	38.8889	0.205244	newly 5 weeks cumul	270.	274.222	{178., 397.}	25.8539	0.113227	newly 58 weeks cumul	460.	377.458	{222., 624.}	32.6661	0.170987
49	newly 3 weeks spec	56.	35.8169	{17., 62.}	44.5926	0.278182	newly 3 weeks cumul	258.	164.549	{91., 255.}	41.4729	0.249285	newly 5 weeks spec	43.	15.7887	{7., 26.}	64.0681	0.495579	newly 5 weeks cumul	341.	204.746	{115., 327.}	44.2278	0.272688	newly 60 weeks cumul	475.	235.732	{127., 375.}	52.5308	0.351271
50	newly 3 weeks spec	49.	7.26984	{1., 16.}	85.1636	1.09653	newly 3 weeks cumul	150.	34.3492	{9., 70.}	77.1005	0.798473	newly 5 weeks spec	26.	3.61905	{0., 10.}	86.0806	1.2086	newly 5 weeks cumul	211.	42.6032	{11., 87.}	79.8089	0.860946	newly 58 weeks cumul	361.	48.6032	{12., 110.}	86.5365	1.04214
